# SO_2_ Solvation in the 1-Ethyl-3-Methylimidazolium Thiocyanate Ionic Liquid by Incorporation into the Extended Cation–Anion Network

**DOI:** 10.1007/s10953-015-0321-5

**Published:** 2015-03-31

**Authors:** Dzmitry S. Firaha, Mikhail Kavalchuk, Barbara Kirchner

**Affiliations:** Mulliken Center for Theoretical Chemistry, Institut für Physikalische und Theoretische Chemie, University of Bonn, Beringstraße 4+6, Bonn, Germany

**Keywords:** Linker effect, Sulfur dioxide, Ionic liquids, Gas absorption, Thiocyanate-SO_2_ adduct, 1-Ethyl-3-methylimidazolium thiocyanate

## Abstract

We have carried out an ab initio molecular dynamics study on the sulfur dioxide (SO_2_) solvation in 1-ethyl-3-methylimidazolium thiocyanate for which we have observed that both cations and anions play an essential role in the solvation of SO_2_. Whereas, the anions tend to form a thiocyanate- and much less often an isothiocyanate-SO_2_ adduct, the cations create a “cage” around SO_2_ with those groups of atoms that donate weak interactions like the alkyl hydrogen atoms as well as the heavy atoms of the $\pi$-system. Despite these similarities between the solvation of SO_2_ and CO_2_ in ionic liquids, an essential difference was observed with respect to the acidic protons. Whereas CO_2_ avoids accepting hydrogen bonds form the acidic hydrogen atoms of the cations, SO_2_ can from O(SO_2_)–H(cation) hydrogen bonds and thus together with the strong anion-adduct it actively integrates in the hydrogen bond network of this particular ionic liquid. The fact that SO_2_ acts in this way was termed a linker effect by us, because the SO_2_ can be situated between cation and anion operating as a linker between them. The particular contacts are the H(cation)$\cdots$O(SO_2_) hydrogen bond and a S(anion)–S(SO_2_) sulfur bridge. Clearly, this observation provides a possible explanation for the question of why the SO_2_ solubility in these ionic liquids is so high.

**Electronic supplementary material:**

The online version of this article (doi:10.1007/s10953-015-0321-5) contains supplementary material, which is available to authorized users.

## Introduction

The capture of sulfur dioxide (SO_2_) has drawn significant attention because SO_2_ is one of the most harmful air pollutants, mainly originating from the combustion of fossil fuels [1]. The most traditional and widely used technology for SO_2_ capture from flue gases is limestone scrubbing [2]. This process has certain disadvantages [3], including irreversibility of the reaction and a large amount of waste. Recently, the absorption of SO_2_ in ionic liquids (ILs) was suggested as an alternative [4]. The high absorption capacity and the good reversibility of the absorption process as well as unique and tunable properties of ILs have caused a growing interest for the past decade [4–8].

SO_2_ absorption by ILs can occur in a physical or in a chemical way [4, 5, 8] from which only in the former case a full and simple recovery is possible. Therefore, the appropriate media for the full or partial recovery of SO_2_ depending on the purpose can be chosen. The physical absorption of SO_2_ in ILs is almost independent of the type of anion and cation [8], whereas in case of the chemical absorption the dependence on the type of IL becomes more pronounced with the nature of the anion playing the crucial role [8–10]. Such a principle difference in the behavior on the microscopic scale might be better understood when theoretical methods are used.

Static gas-phase calculations of interaction energies and the assigment of principle interaction types based on those values were investigated in several articles [10–13]. Also, molecular dynamics simulations employing empirical force fields were used for evaluating some physical properties of the systems [14–17]. In order to understand the mechanism of SO_2_ solvation in more detail, solute–solvent interactions should be taken into account. Thus, the investigation of specific interactions between SO_2_ and the IL components, as well as understanding how the interactions influence the conditions in the IL, can be provided from a valuable theoretical background which aids in the design of ILs with desired properties.

In this work, we have employed ab initio molecular dynamics (AIMD) simulations to obtain insight into the structural and dynamic properties of the SO_2_–IL systems. To do so, we have chosen 1-ethyl-3-methylimidazolium thiocyanate ([$\hbox {C}_2\hbox {C}_1\hbox {Im}$][SCN]) as a model system. This IL is one of the most promising candidate for large-scale application, possessing one of the highest capacities of SO_2_ absorption, a rapid absorption rate, and excellent reversibility [12]. Moreover, the solubilities of other gases in this IL are significantly lower than for SO_2_ (comparing the molar fraction of gases in [$\hbox {C}_2\hbox {C}_1\hbox {Im}$][SCN] at 1 bar and 293 K: SO_2_ is 75 % [12], $\hbox {NH}_3$—16.3 % [18], CO_2_—1.2 % [19]), which is a requirement for separation processes.

Recently the structural properties of pure [$\hbox {C}_2\hbox {C}_1\hbox {Im}$][SCN] [20, 21] and its mixtures with another ionic liquid [22], as well as carbon dioxide (CO_2_) absorption in imidazolium and ethylammonium ionic liquids [23–25], have been studied from AIMD, providing a solid background for the current investigation. Thus, the knowledge gathered here provides a more thorough understanding of SO_2_ solvation in ILs. Moreover, similarities and differences can be identified between SO_2_ and CO_2_ with respect to their solvation in ILs.

## Computational Details

**Fig. 1:**
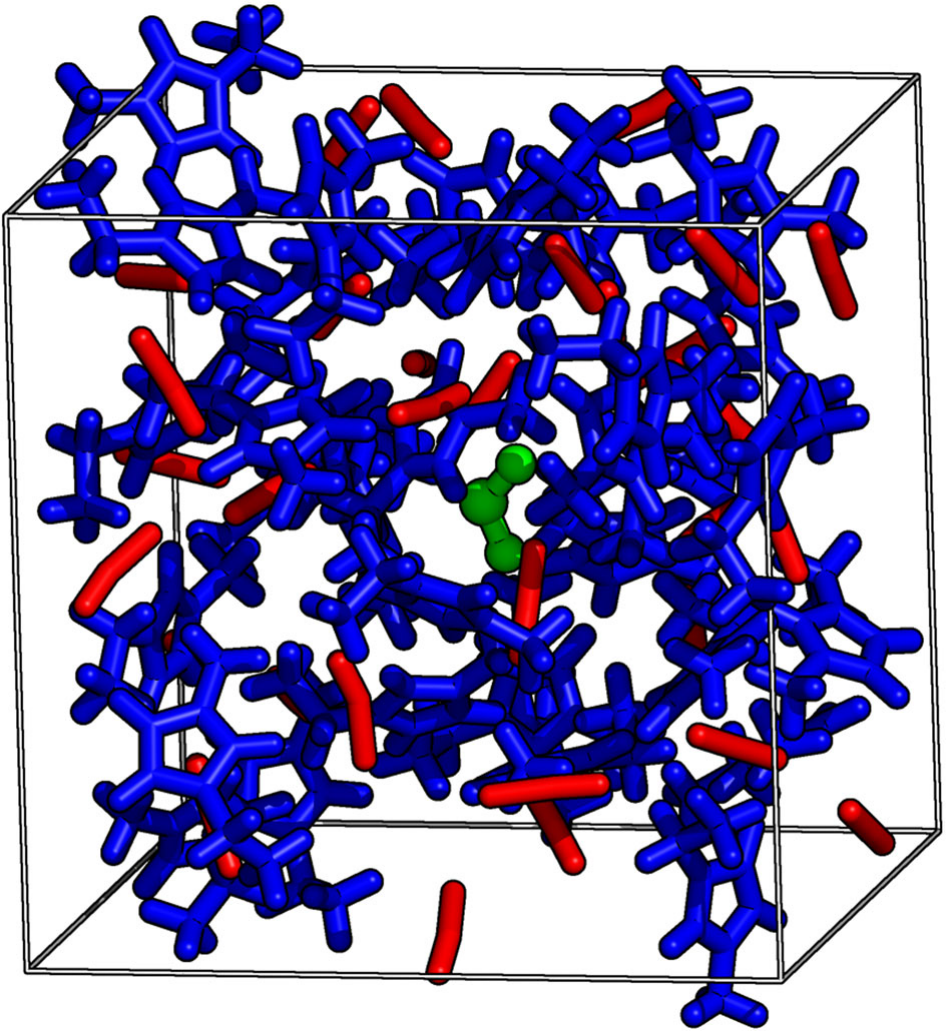
Representative snapshot of the simulation boxes: 1-ethyl-3-methylimidazolium thiocyanate in stick (cations in* blue*, anions in* red*) and SO_2_ (in* green*) in* ball-and-stick* representation

The mixture of [$\hbox {C}_2\hbox {C}_1\hbox {Im}$][SCN] with SO_2_ (32:1 molar ratio) was studied from AIMD. A system containing 32 ion pairs of [$\hbox {C}_2\hbox {C}_1\hbox {Im}$][SCN] and one SO_2_ molecule was simulated in a cubic box with a size of 2031.4 pm (this corresponds to $\rho =1.086\hbox { g}{\cdot}{\text{cm}}^{-3}$) with periodic boundary conditions (Fig. 1). For details on the preparation of the starting geometry for the system, see the supporting information.

The AIMD simulations were carried out with the CP2k [26] program package, using the Quickstep module [27] with the orbital transformation method for faster convergence [28]. The electronic structure was calculated employing the density functional theory utilizing the BLYP-D3 functional with the empirical dispersion correction (D3 with zero dumping) from Grimme [29], since the dispersion-corrected exchange-correlation functional has provided reasonable results for ionic liquids [20, 30–32]. The molecularly optimized double-$\zeta$ basis set (MOLOPT-DZVP-SR-GTH) [33] with corresponding Goedecker–Teter–Hutter pseudopotentials [34–36] was applied for all atoms. The density smoothing for the electron density (NN10_SMOOTH) and its derivative (NN10) was used [27]. The CUTOFF criterion for the finest grid level for the DFT calculations was 300 Ry.

The temperature was thermostated to 350 K by Nosé–Hoover chain thermostats [37–39] with a time constant of 100 fs for individual atoms for a total of 5.0 ps and for the complete system in the main run. For the equilibration (5.0 ps) the time step 0.5 fs was used. Since this value provided a high energy drift ($2.8 \times 10^{-5}\hbox { a.u.}{\cdot}\hbox {fs}^{-1}$) in the beginning of the main run (first 22.3 ps), a shorter time step (0.25 fs) was applied. This decreased the energy drift by one order to $3.7 \times 10^{-6}\hbox { a.u.}{\cdot}\hbox {fs}^{-1}$) , and we excluded the first 25.0 ps (22.3 with 0.5 and 2.7 ps with 0.25 fs time steps) of the main run from further consideration. The production simulation was subsequently run for 58 ps.

**Fig. 2:**
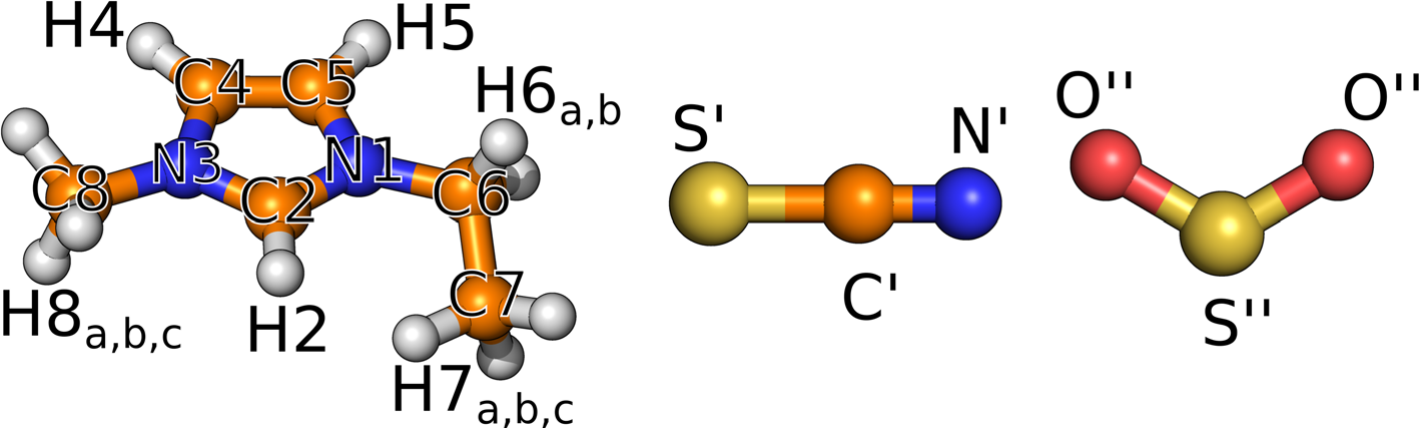
Ball-and-stick representation of the cation, the anion, and the SO_2_ molecule. N:* blue*; C:* orange*; O:* red*, H:* white* and S:* yellow*. (Please note that the cation’s atoms are marked* without primes*, the anion with* single primes*, and the atoms of the SO_2_ with* double primes*.)

Static quantum chemical calculations were performed from the density functional theory (DFT) and wave function theory with the ORCA [40] program (version 3.0.0 [41]). Geometry optimization was performed on the B3LYP-D3(BJ)/def2-TZVPP level, whereas the final energy calculations were done based on DFT geometry applying the CCSD(T) level of theory. Extrapolation to the complete basis set limit was carried out according to a two-point extrapolation scheme separately for Hartree–Fock energies and CCSD(T) correlation energies. The calculation of SO_2_ gas frequencies was performed on BLYP-D3(BJ)/def2-TZVPP in order to be consistent with the exchange correlation functional, which was applied for bulk simulation. Structural analyses of the trajectory were performed using TRAVIS [42, 43]. Molecule representations were visualized using PyMol [44], and all graphs were created using Gnuplot 4.6. [45] The atom labeling used in the following discussion is shown in Fig. 2.

## Results and Discussion

### Cation–Anion and Cation–SO_2_ Interactions

To gain insight into how SO_2_ in low concentrations influences the structure of [$\hbox {C}_2\hbox {C}_1\hbox {Im}$][SCN], we compared corresponding radial distribution functions (RDFs) of the pure IL to the system under study (see supporting information Figs. S1–S2 and Ref. [20]). Significant changes in the structure of the IL were not found. This is consistent with MD simulations of systems with a higher concentration of SO_2_ in ILs [10, 17] as well as with the results obtained previously for CO_2_ in ethylammonium nitrate [25] and in 1-ethyl-3-methylimidazolium acetate [23, 24]. To reveal how SO_2_ enters the IL structure and to characterize the solvation shell of the solute molecule, we considered RDFs as well as Voronoi analysis. The latter analysis provides valuable information about the time development or the average of surface covering of a certain particle by other particles or groups of atoms.

**Fig. 3:**
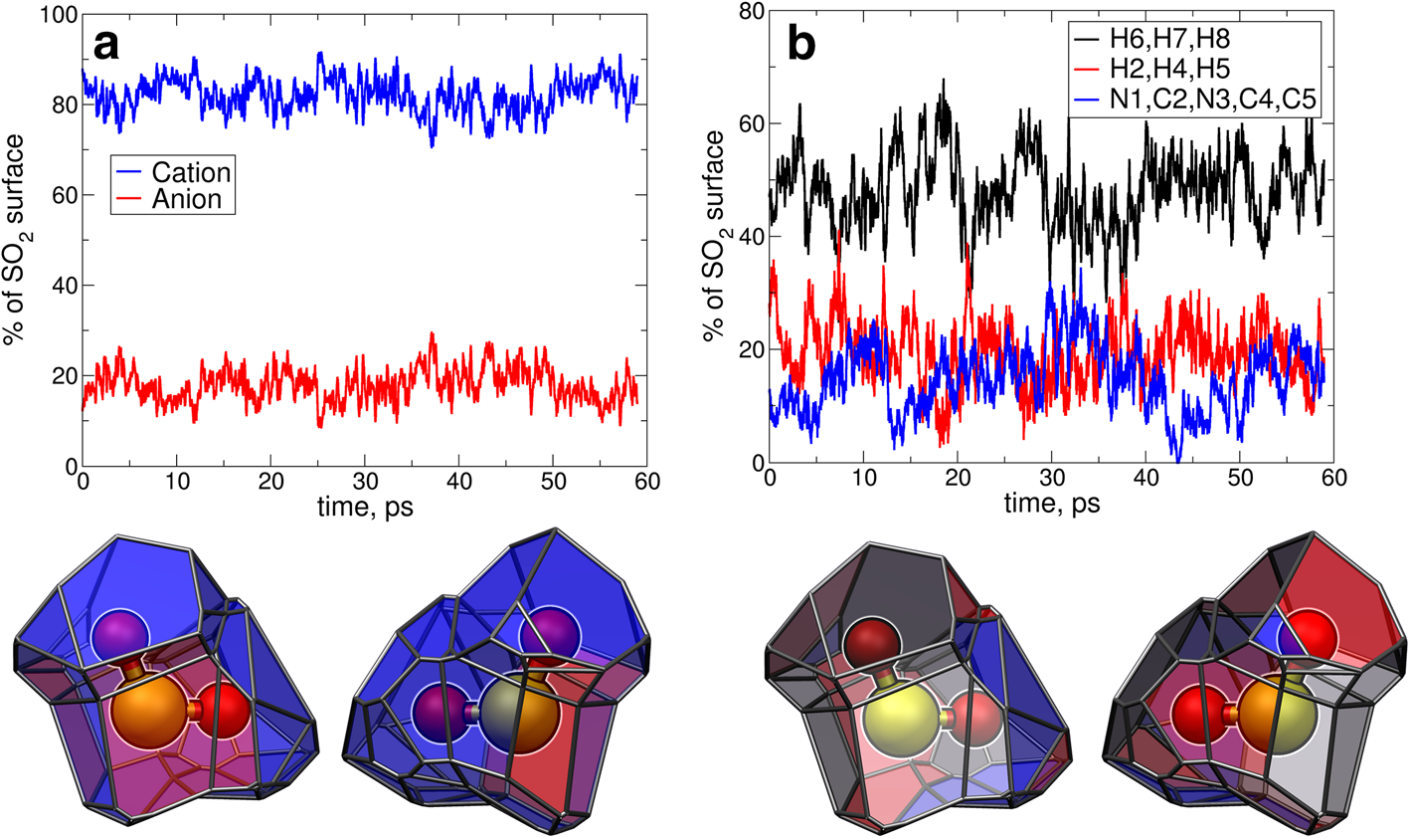
*Top* time-development of the SO_2_ surface covering by ions (**a**, *blue lines* are cation atoms, and* red lines* are anion atoms) and cation atoms (**b**, *black lines* are alkyl hydrogen atoms,* red lines* are ring hydrogen atoms and* blue lines* are ring atoms).* Bottom* representative SO_2_ Voronoi surfaces, color code for the surfaces is similar with those for graphs above. (Note* grey* color was used for the segments belonging to the anion for the* bottom right* Voronoi surfaces)

Similar to CO_2_ in 1-ethyl-3-methylimidazolium acetate [23, 24], SO_2_ is surrounded only by one anion and five cations on average in the first solvation shell (the numbers were defined based on the value of integral in the first minimum of the corresponding RDF between centers of mass of solute and ions, see Fig. S3 in the supporting information). From Fig. 3a it is apparent that a similar ratio of anions to cations is obtained from the average SO_2_ surface covering by anions and cations (18 vs. 82 %). Both results — RDF as well as Voronoi — indicate the presence of a “cation cage” around SO_2_.

Since a relatively large number of cations was detected in the first solvent shell of SO_2_, it is worth determining in detail which functional groups are important, and how this situation of SO_2_ solvation compares to the CO_2_ solvation by ILs. To reveal the role of the specific interactions, especially the weak interactions, in the SO_2_ solvation, we have separated the SO_2_ surface coverage from the cations into three groups: ring hydrogen atoms (H2, H4, H5), heavy ring atoms (N1, C2, N3, C4, C5), and alkyl hydrogen atoms (H6, H7, H8). For these groups we have plotted, in Fig. 3b, their fraction of the coverage of the Voronoi surface. It is a reasonable first approximation that a larger coverage (i.e., closest neighbor) corresponds to a more important role in the solute solvation. The ring hydrogen atoms possess a relatively strong interaction with the solute but also with the anion. The strong nature of the latter interaction was shown in several quantum chemical studies [46, 47]. If there are contacts between the SO_2_ and the cation ring other than via the ring hydrogen atoms, these are most likely weak solute–$\pi$–system interactions. Also the alkyl hydrogen atom contacts to the solute represents weak dispersion interaction [46, 47]. Interestingly, the alkyl hydrogen atoms (48 %) and heavy ring atoms (15 %) coverages, corresponding to the weak interactions, dominate over the coverage of the ring hydrogen atoms (19 %) which correspond to strong interactions. Moreover, these portions are comparable with those from the non-polar part of ethylammonium nitrate to CO_2_ solvation [25]. From these results it is apparent that weak interactions are important not only for the solvation of CO_2_ [23–25] but also for the solvation of SO_2_. To support this observation, we compared the experimental SO_2_ solubility in different ionic liquids. The increase of the cation’s side chain results in the increase of the SO_2_ solubility [5, 10, 48] which agrees with our findings.

**Fig. 4:**
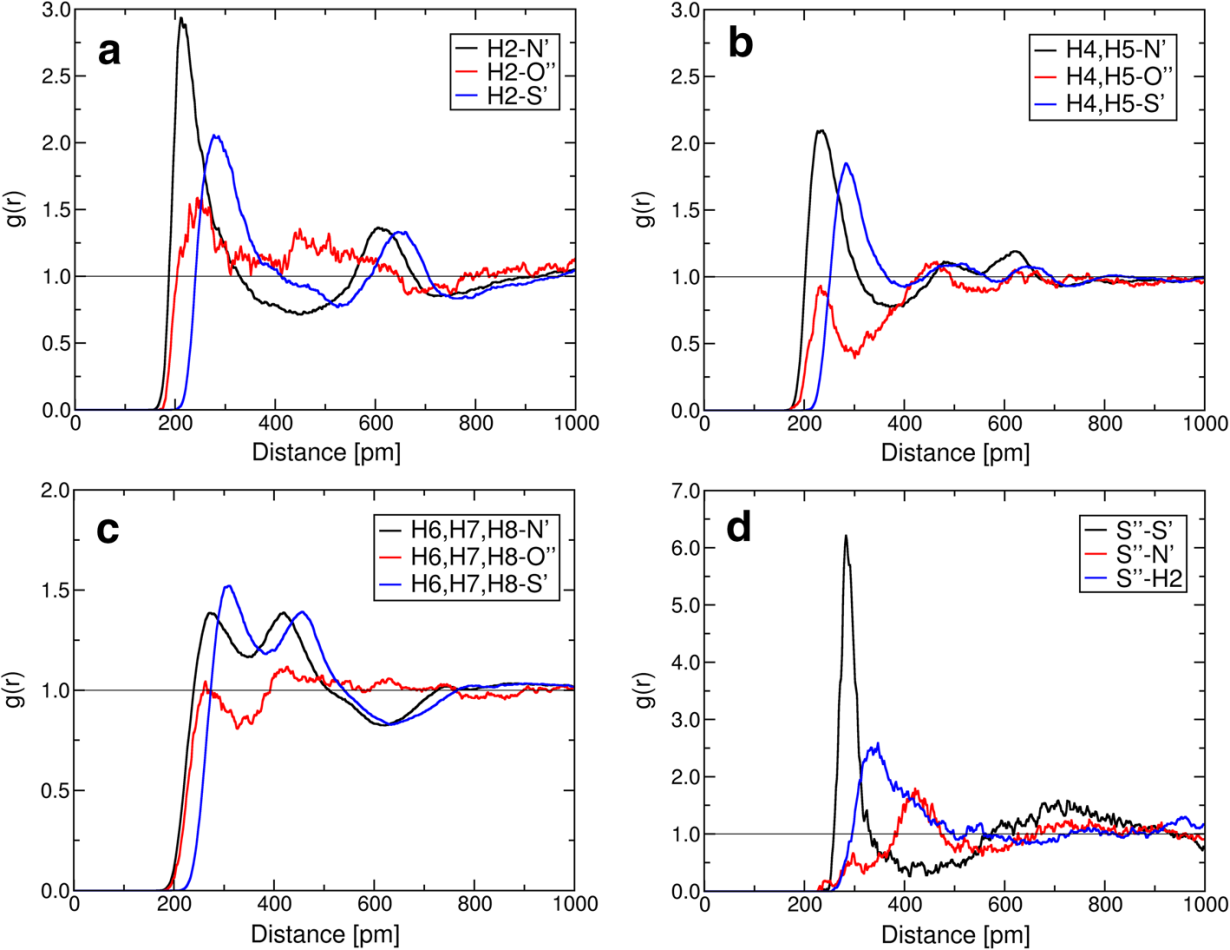
Cation–anion and ion–SO_2_ interactions. The RDFs between hydrogen atoms and selected atoms form anion and SO_2_ are presented on **a**–**c**, whereas ** d** contains the RDFs between S″ (from SO_2_) and selected atoms from cation and anion

The RDFs, which reflect probabilities of finding two atoms at certain distances normalized by the density, of the cation’s hydrogen atoms (H2, H4–H5, H6–H8) with the oxygen atoms of the sulfur dioxide (O″) and the anion tail atoms (N′ and S′), are presented in Fig. 4 on a–c. There is a noticeable similarity in the position of the first maximum for the cation-N′ and cation-O″ functions albeit with the difference that the O″(SO_2_) peaks are less pronounced than the N′($[\hbox {SCN}]^-$) peaks. Remarkably, the interplay of CO_2_ with a cation in ethylammonium nitrate [25] or in 1-ethyl-3-methylimidazolium acetate [23, 24] shows the opposite behavior, i.e., there are no such peaks between the oxygen atoms of CO_2_ and the acidic hydrogen atoms of the cation. Thus, there is no contact of a CO_2_ with the cation via the acidic hydrogen atoms. The CO_2_ solvation rather takes the form that it competes with dispersion forces in the system such as anion–cation side chain, $\pi$–$\pi$ stacking, and side chain-side chain [46, 47] interactions. These essential differences in the structure of solvated SO_2_ and the solvated CO_2_ might be one of the reasons for the significantly higher solubility of the former gas in ILs.

Since not only the nature of the anion and the side chain of the cation are important for the SO_2_ solvation but also the role of acidic hydrogen atoms, we have examined how the cation exchange in principle influences the SO_2_ solubility. For example, the experimental findings on the cation exchange of 1-alkylpyridinium to 1-alkyl-3-methylimidazolium show a slight decrease in the SO_2_ solubility in ILs with chloride, bistriflimide, and tetrafluoroborate anions [10, 11]. This changes significantly when ILs with a thiocyanate anion are considered. When the 1-butylpyridinium cation is exchanged for the 1-ethyl-3-methylimidazolium cation, both with the thiocyanate anion, the SO_2_ solubility increases from 2.6 to 3.0 mol per one mol IL (at 0.1 MPa and 293 K) [10, 12]. These contrasting experimental observations might be related to different SO_2_ solvation mechanisms which can be explained by microscopic insight given for example by simulations, i.e., in the 1-ethyl-3-methylimidazolium thiocyanate ionic liquid we have detected frequent conformations in which the ring hydrogen atoms of the cations are close to the oxygen atoms of the SO_2_ and the sulfur atoms of the anions are close to the sulfur atom of the SO_2_. It is likely that this newly observed conformation, which we have termed linker conformation or linker effect, is responsible for the good incorporation of SO_2_ in this particular ionic liquid by the formation of the following linked structure H2, H4, H5(cation)$\cdots$O″(SO_2_)–S″(SO_2_)$\cdots$S′(anion).

Possible linker bonds between ring hydrogen atoms of the 1-butylpyridinium cation and SO_2_ should be weaker due to the lower acidity compared with those for the 1-ethyl-3-methylimidazolium cation. Thus, the incorporation of SO_2_ into the network of hydrogen bonds is limited in the case of ILs consisting of 1-butylpyridinium cations.

### Anion–SO_2_ Interactions

**Fig. 5:**
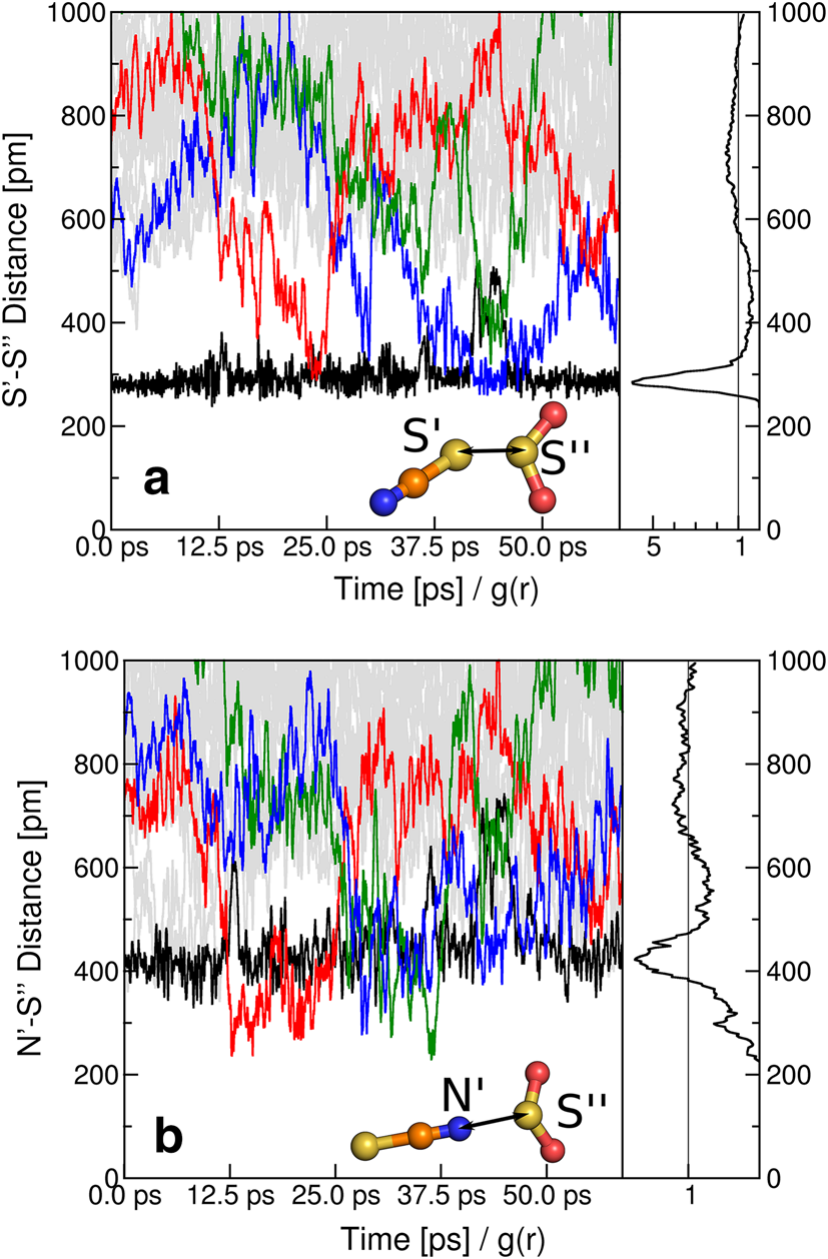
Anion–SO_2_ interaction. Time development of distances between S″ atom of SO_2_ and S′ (A) or N′ (B) atoms of all 32 anions.* Curves* corresponding to anions that approach the SO_2_ molecule closer than 350 pm during the simulation are colored *black*, *red*, *blue* and *green*. *Curves* corresponding to other anions are colored* grey*

Only one anion was found in the first solvation shell of SO_2_. To understand the nature of the intermolecular forces between the anion and the SO_2_, we have carried out static quantum chemical calculations regarding the formation of the anion–SO_2_ complex. The results from these calculations showed comparable interaction energies for possible adducts ($-68.6\, \hbox {kJ}{\cdot}\hbox {mol}^{-1}$ for the thiocyanate-SO_2_ adduct $[\hbox {NCS}{\cdot}\hbox {SO}_2]^-$ and $-65.7\,\hbox {kJ}{\cdot}\hbox {mol}^{-1}$ for the isothiocyanate-SO_2_ adduct $[\hbox {SCN}{\cdot}\hbox {SO}_2]^-$). Thus, it is likely that the formation of both complexes might occur during the simulation with almost equal probability. Nevertheless, the RDFs for S′–S″ and N′–S″ distances indicate the formation of the thiocyanate adduct is dominant in the system under investigation as shown in Fig. 4d. The time development of the distances between the S″ atom and the S′ or N′ atoms of all anions, as shown in Fig. 5a and b, indicates that SO_2_ interacts with one [SCN]$^-$ over the majority of the simulation time, see in Fig. 5a, black line. However, a temporary anion exchange (red, blue and green lines in Fig. 5a) is observed at around 23 ps and between 45 and 48 ps. The substitution of the S′ atom to the N′ atom coordination or the change from thiocyanate-SO_2_ to isothiocyanate-SO_2_ aducct occurs at around 12, 14, and 36 ps (see Fig. 5b, red, blue and green lines). This transition in the anion coordination at SO_2_ is in good agreement with the experimental results of the thiocyanate anion complex formation with SO_2_ in dilute solutions of acetonitrile [49]. Thus, the absorption of SO_2_ in the ionic liquid under consideration does not take place with a pure thiocyanate-SO_2_ complex generation, rather an equilibrium mixture of thiocyanate and isothiocyanate adducts will form.

**Fig. 6:**
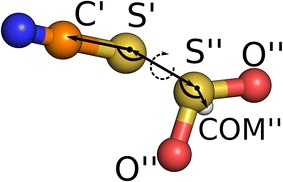
*Ball-and-stick* representation of $[\hbox {NCS}{\cdot}\hbox {SO}_2]^-$ complex. For structural parameters see Table 1

To understand the structure of the dominant thiocyanate-SO_2_ adduct in more detail, we have compared the geometrical parameters from AIMD simulation in bulk and from the static quantum chemical calculation of the isolated adducts with data from crystal structures of the potassium 1,4,7,10,13,16-hexaoxacyclooctadecane thiocyanate-SO_2_ adduct [K(18-crown-6)][$\hbox {NCS}{\cdot}\hbox {SO}_2$] [50] and the tetramethylammonium thiocyanate-SO_2_ adduct $[\hbox {NMe}_4][\hbox {NCS}{\cdot}\hbox {SO}_2]$ [51] (see Fig. 6 and Table 1). Both calculated structures agree well with the crystallographic data for the thiocyanate-SO_2_ adducts, see Table 1. The differences in distances and angles are explained by the different surroundings for $[\hbox {NCS}{\cdot}\hbox {SO}_2]^-$, particularly, the C′-S′-S″-COM″ dihedral angle could be smaller in absolute values due to the stabilization of the selected structure. Considering the most probable distance between S′ and S″ atoms (283 pm), we also found good agreement with experimental values of S$\cdots$O distances in complexes of SO_2_ with diethyl ester (287 pm) and $\hbox {H}_2\hbox {O}$ (282 pm) [52].

**Table 1:** Structural parameters for the thiocyanate-SO_2_ adduct

	Bulk^a^	Gas^b^	[K(18-crown-6)]$[\hbox {NCS}{\cdot }\hbox {SO}_2]^{\rm c}$	$[\hbox {NMe}_4][\hbox {NCS}{\cdot}\hbox {SO}_2]^{\rm c}$
S′–S″ (pm)	283	271	274	301
O″–S″–O″ (°)	116	115	119	115
C′–S′–S″ (°)	96	97	99	90
S′–S″–COM″ (°)	109	112	103	107
C′–S′–S″–COM″ (°)	$\pm$142	139	−91	74

^a^The maximum of corresponding distribution function from $\mathbf{32}\mathbf{P}+\mathbf{SO}_\mathbf{2}$ trajectory (see supporting information Figs. S4–S6)

^b^The structure was optimized in the gas-phase on B3LYP-D3/def2-TZVPP level of theory

^c^The distance and angles were evaluated from crystallographic structure from Refs. [50] and [51]

**Fig. 7:**
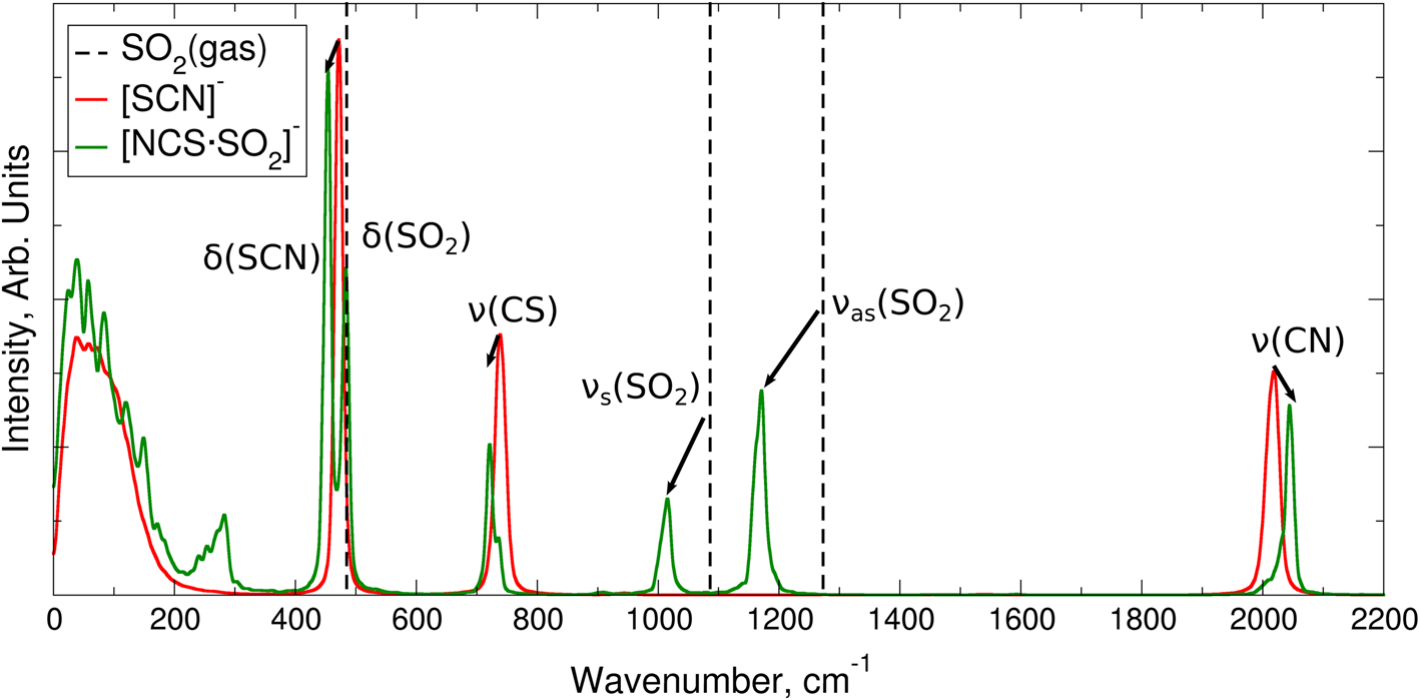
Power spectra 31 anions (*red*) and thiocyanate-SO_2_ adduct (*green*). *Vertical dashed black lines* mark SO_2_ frequencies from gas-phase calculation on the BLYP-D3(BJ)/def2-TZVPP level of theory

To clarify how the vibrational frequencies of the SO_2_ and the thiocyanate anion change upon thiocyanate-SO_2_ adduct formation, power spectra for the adduct have been calculated and compared with the gas-phase vibrational frequencies for SO_2_ and the power spectra for the remaining anions (Fig. 7). Moreover, we summarize the information on the calculated and experimental data for unbound (thiocyanate in bulk and SO_2_ in gas-phase) and bound states ($[\hbox {NCS}{\cdot}\hbox {SO}_2]^-$) of the SO_2_ and the thiocyanate anion (Table 2). It is apparent that the direction of relative shifts for the bands (blue or red shift) are in good agreement with the experimental results, whereas the absolute position of the maximum for the absorption band is not reproduced satisfactorily due to the deficiency of the BLYP functional, as has been oberserved previously [43]. Interestingly, the experimental infrared spectra for SO_2_ dissolved in thiocyanate ILs [10, 12] indicate that the absorption bands of dissolved SO_2_ are almost at the same position as the fundamental frequencies of SO_2_ in the gas-phase [53]. Care has to be taken in interpretation of the total spectra since some of the adduct bands might overlap with other bands or have low intensity compared to the rest of unbound SO_2_.

**Table 2:** Vibrational frequencies in cm^−1^ for SO_2_, thiocyanate and thiocyanate-SO_2_ adducts

	Calc.		Expt.	
	SO_2_ (gas), $[\hbox {SCN}]^-$(bulk)	$[\hbox {NCS}{\cdot}\hbox {SO}_2]^-$	SO_2_ (gas), $[\hbox {SCN}]^-$(bulk)	$[\hbox {NCS}{\cdot}\hbox {SO}_2]^-$ or $[\hbox {NC}{\cdot}\hbox {SO}_2]^-$
$\nu$(CN)	2019	2044	2052 [50]	2090 [50]
$\nu _{as}(\hbox {SO}_2)$	1273	1171	1362 [53]	1152^a^ [54]
$\nu _{sym}(\hbox {SO}_2)$	1086	1015	1151 [53]	1107 [50] (1065^a^ [54])
$\nu$(CS)	739	722	732 [50]	725 [50]
$\delta (\hbox {SO}_2)$	485	484	528 [53]	–
$\delta$(SCN)	472	454	–	–

^a^The data were taken for the cyanide-SO_2_ adduct

## Conclusion

Obtaining a picture, with microscopic resolution, of the solvation of small gas molecules (like SO_2_) is crucial to understanding the varying solubilities in different ionic liquids. In this article we have provided a detailed investigation of SO_2_ solvation in the [$\hbox {C}_2\hbox {C}_1\hbox {im}$][SCN] ionic liquid from AIMD. This system is known to be special, because of its high SO_2_ solubility. Contacts between the SO_2_ and groups that donate weak interactions, like the alkyl hydrogen atoms as well as the $\pi$-system of the cation, are numerous in the first solvent shell, whereas only one single thiocyanate anion is found in the first solvent shell forming an anion-SO_2_ complex. The dynamics of the anion exchange at the SO_2_ was investigated and the formation of different anion-SO_2_ adducts were detected. The geometry of the most probable thiocyanate-SO_2_ adduct of our bulk simulations resembles those from the static gas-phase calculations and from the available crystal structure, namely we find a pronounced sulfur–sulfur bridge between the anion and the SO_2_ which in a few instances is replaced by a N(anion)–S(SO_2_) isothiocynate-adduct. The qualitative and quantitative agreements between calculated and experimental frequencies, as well as potentially important bands for identification of the absorption of SO_2_, were detected. More interestingly, and in clear contrast to CO_2_, we observed that SO_2_ is capable of forming a hydrogen bond with the acidic ring protons of the cation. Thus instead of showing only the usual solvation pattern, we observed that the SO_2_ molecule is incorporated into the ionic liquid network, a contact which we called a linker effect, i.e., SO_2_ can interact strongly with both the cation and the anion at the same time. Undoubtly, this linker effect plays a crucial role in the high solubility of SO_2_ in the [$\hbox {C}_2\hbox {C}_1\hbox {Im}$][SCN] IL and does not occur in the solvation of CO_2_. It was previously found that while CO_2_ interacts with the anions of ILs, it does not form hydrogen bonds by accepting the acidic protons of either the imidazolium or the ammonium cation. Therefore, CO_2_ it is not incorporated in the hydrogen bonding network of the ionic liquid. On the contrary, for the solvation of SO_2_ in the [$\hbox {C}_2\hbox {C}_1\hbox {im}$][SCN] ionic liquid, a network of H(cation)$\cdots \hbox {O}(\hbox {SO}_2) {-}{\hbox {S}}(\hbox {SO}_2)\cdots \hbox {S}$(anion), with SO_2_ being a linker molecule, can be fabricated. This means, that given the right combination of cation and anion where the SO_2_ is able to form hydrogen bonds with the cation and a sulfur–sulfur bridge (or a similar bond leading to a strong adduct) with the anion, a good solubility of the SO_2_ in the IL should be observed. Thus, the design of potential ionic liquids containing good hydrogen bond donor ability in the cation and sulfur atoms free to from sulfur–sulfur bridges or similar strong adducts in the anion should not only lead to a specific absorption of this particular gas molecule, but also to the application of particular purposes of this gas-IL mixture where a more extended hydrogen bond network is needed.

## Electronic supplementary material

Below is the link to the electronic supplementary material.

Supplementary material 1 (pdf 444 KB)
