# Supplementary material for: SO2 Solvation in the 1-Ethyl-3-Methylimidazolium Thiocyanate Ionic Liquid by Incorporation into the Extended Cation–Anion Network
Source: J Solution Chem. 2015 Mar 31;44(3):838–49. doi: 10.1007/s10953-015-0321-5 (PMC4412831; doi:10.1007/s10953-015-0321-5)
Supplement: Supplementary file 1 — Supplementary material 1 (pdf 444 KB) [file 10953_2015_321_MOESM1_ESM.pdf]

Supporting Information

SO2 Solvation in the 1-Ethyl-3-Methylimidazolium Thiocyanate Ionic Liquid by Incorporation into the Extended Cation - Anion Network

Dzmitry S. Firaha[a], Mikhail Kavalchuk[a], Barbara Kirchner[a*]

[a] *Mulliken Center for Theoretical Chemistry, Institut für Physikalische und Theoretische Chemie, University of Bonn, Beringstr. 4+6, D-53115 Bonn, kirchner@thch.uni-bonn.de*

The box size for the simulated systems were calculated based on the assumption of ideal mixing. The density for pure 1-ethyl-3-methylimidazolium thiocyanate at 350 K was interpolated from experimental data between 278 and 363 K (J. Chem. Eng. Data, **2011**, *56*, 4813), whereas the density for liquid SO_2 (at 350K and 16.72 bar) was taken from Thermophysical Properties of Fluid Systems on the http://webbook.nist.gov/chemistry/fluid/. Since the isothermal compressibility of liquids is approximately 10^{-5} bar^{-1}, we neglect the volume expansion for SO_2.

The starting geometry for the simulation of 32P+SO_2 system

C -8.426095529 -10.447671791 1.662448252
C -13.862827864 -11.973591752 -10.854858996
C -9.675069569 -18.076239194 6.358246426
C -7.214913632 -10.883281748 -12.892760464
C -10.973198579 -22.398034343 -13.674221122
C -11.001924656 -17.892800049 2.242669524
C -12.711781689 -17.740369182 -7.624860347
C -7.619083296 -23.595906355 4.589887186
C -10.003928415 -23.957525179 0.292197631
C -12.733523057 -5.23294725 -7.876645105
C -9.034317658 -23.937097138 2.520287927
C -0.990349592 -25.9036492 0.543405655
C -8.971449571 -11.25441706 -0.569343585
C -11.421535189 -6.902011289 -3.896105308
C -6.609472112 -13.638142962 -2.060809157
C -9.498165901 -1.860803498 11.362767702
C -6.780162443 -24.847474813 4.203634357
C -9.890716404 -13.345499032 -11.631807948
C -9.497863071 -19.556560669 8.500354126
C -10.160182875 -7.245332076 -5.635416108
C -8.526531375 -20.612826005 8.075078718
C -6.650743326 -12.077960725 -12.010034414
C -5.959511506 -24.034359316 0.64416085
C -9.731621842 -12.859057497 -5.377170978
C -0.852784677 -26.261340151 4.090390938
C -4.370907469 -24.015627126 0.784845567
C -7.959346368 -9.192793064 -7.135374808
C -9.167446963 -14.804364843 -7.003254701
C -5.499905334 -10.481429135 -10.344605499
C -9.881457091 -13.499295701 -4.004433506
C -8.423039301 -10.10448709 -1.442471025
C -8.008459899 -15.397972622 -7.387351894
C -10.973473769 -5.722007033 -4.336171027
C -0.059686916 -26.239502461 1.734373482
C -2.533185479 -13.435473781 -1.579759603
C -3.582703763 -25.084786887 -1.338505952
C -1.437376898 -27.177137045 4.900385836
C -4.388264022 -7.817980365 7.714135127
C -6.669421089 -12.239110751 -9.578039001
C -2.119741127 -19.212930671 1.493757578
C -4.965154497 -9.279602555 -8.264971107
C -7.487597156 -13.512671198 -6.270239216
C -4.090509362 -14.396041528 3.424262942
C -5.536725066 -14.805027184 -7.239847966
C -6.31550215 -11.474612681 -8.459115038
C -2.639407381 -24.846556186 -2.258287998
C -9.528193784 -5.053850702 -6.322476609
C -3.257144008 -19.864309948 0.65279798
C -1.421859858 -28.082464683 2.846765523
C -4.549158335 -18.062546111 -0.777529959
C -2.656403972 -23.140076141 -0.772607061
C -4.354972604 -8.591237402 -3.376182821
C -0.999818549 -15.146306167 -2.744537093
C 1.162998864 -17.510969161 1.109498729

C 0.07386603 -16.626101502 0.43371634
C -5.601350455 -26.145043815 -6.58429477
C -5.321717989 -21.191125492 3.925418448
C -3.896416584 -7.483219327 -4.343499925
C -1.418585575 -21.941412134 4.959106463
C -8.812911052 -18.835208889 -7.622474035
C -3.101169502 -14.905948708 -3.460622584
C -4.950002048 -18.610193981 -7.576830138
C 1.242931666 -11.6870855 9.667257601
C -5.618166908 -17.207023242 -0.552714556
C -4.186491848 -7.981994218 1.714772427
C -2.459386556 -29.485345233 4.731884268
C -2.83776921 -13.42308178 6.780386613
C -5.611136869 -8.102398613 -1.041831618
C -1.129144053 -15.81659698 -3.929147353
C -3.639583798 -11.873394755 1.015166073
C -0.34126582 -11.8418568 7.73143852
C 1.057474266 -14.090944867 2.816063183
C -3.441260122 -23.600747053 5.464833152
C -5.367018208 -18.651794053 1.178924111
C -0.930635557 -22.898585624 -2.544399943
C -1.54710566 -12.603128297 2.136982773
C 1.434030735 -4.174414481 -10.470094073
C -6.690550948 -7.363550679 -0.742450907
C -2.443285695 -11.9679202 6.382650146
C 3.455528961 -18.534526667 0.196916346
C -6.611291181 -7.423000397 -2.97579338
C -1.293064906 -11.127012172 9.669699293
C -2.23252411 -11.130361317 8.603895033
C 1.429813809 -19.07331362 -0.777114951
C -2.952849679 -16.365536799 -5.62127571
C -1.567280118 -23.838313467 6.700550152
C -4.948504591 -21.118273589 -8.129344541
C 0.920848799 -12.628171771 2.880933046
C -4.671627719 -15.371239953 9.768781158
C -3.694239837 -24.599755041 6.38251472
C -5.549675688 -20.590233718 -6.014483285
C -7.607314326 -17.281965481 1.142253003
C 1.101728298 -9.788995716 4.62370273
C -1.383455847 -10.813883632 0.731917437
C 3.631658333 -19.579500948 -0.663589794
C -2.117244653 -19.458740947 -3.270328957
C -0.164905285 -11.185442319 1.098970775
C -5.301055283 -22.296006462 -7.446933463
C -2.396155705 -15.99792519 -6.947930793
C -8.48322289 -5.928696991 -1.938493896
C -7.990297618 -3.825508324 11.798621375
C -14.803142663 -12.920643408 -10.847022574
C -9.134375573 -21.869476458 1.69994707
C -8.418108319 -21.850289954 2.812329062
C -14.111237488 -19.717791529 -8.192356582
C -13.036749395 -16.067988172 -11.074130332
C -12.521535636 -20.964973541 -3.999429121
C -11.175264563 -9.384469785 -4.67573506
C -9.132070344 -3.792698168 9.915313269
C -11.827933335 -9.925234399 -9.802190279
C -7.411379558 -12.448070995 1.296976155
C -11.921214006 -11.360652735 -9.230091161
C -9.212313705 -16.861343248 6.05898978
C -8.460317607 -17.288832602 8.128459452
C -12.259747827 -20.290161516 -7.1492078
C -7.939167984 -1.548295343 -1.933654294
C -16.570049889 -21.254455476 -8.539446486
C -14.208496584 -25.920516564 -11.15864877
C -13.888766508 -23.737074287 -1.13431203
C -7.683615198 -4.985830358 11.141057681
C -12.067451725 -15.300992038 -13.214447168
C -12.844540897 -24.800582517 3.59158866
C -12.204149989 -20.517729183 -2.57089951
C -6.914844359 -11.935519724 2.457486357
C -12.966563302 -21.47705905 -7.350404188
C -13.587430715 -23.441981427 -11.332680295
C -7.214718193 -9.766883842 3.741380373
C -11.250419334 -9.961905543 -6.162679751
C -15.239552992 -21.94223566 -8.432146308
C -13.756840201 -13.089722879 -8.931764061
C -13.271498952 -25.41183682 2.455273449
C -14.040573423 -23.028855007 5.132547185
C -13.537404506 -26.476877296 -12.432694655
C -14.223927898 -22.197797058 -11.664537076
C -12.208431963 -18.112224661 -3.300917283
C -15.865203344 -14.445212534 -8.918844376
C -9.902776566 -7.244240311 1.870070051
C -8.628399816 -6.093588333 9.047563443
C -13.033498404 -12.948542678 -5.12099569
C -14.255454777 -15.695379883 -13.265594505
C -11.675031985 -14.331339592 -15.576239107
C -18.13065933 -14.822347904 -1.825283441
C -12.489469804 -13.384783382 -16.445140801
C -7.391881517 -15.372657954 7.012826386
C -15.729418405 -23.978517001 -11.691164634
C -14.952062406 -24.108459132 3.1175901
C -13.835933903 -15.417968671 -14.544665519
C -11.218471563 -17.190868561 -3.406589608
C -20.062252606 -11.06827767 -4.937226988
C -10.705538277 -15.272060528 -0.698270444
C -10.019978171 -6.728719551 9.246462615
C -17.352778723 -14.088416954 -5.646523309
C -10.256067247 -18.923094885 -2.444325328
C -13.09311301 -19.326936293 4.849135215
C -15.408943772 -25.6653518 1.204011671
C -18.135252365 -11.338206991 -3.20218242
C -16.748512288 -21.808720256 -12.192654808
C -15.743529046 -18.333872965 -11.270042698
C -14.36948261 -3.601914408 -4.857980157
C -10.578102501 -14.659826372 0.64938438
C -15.352397703 -7.802241012 -4.411864117
C -12.820892773 -10.624223505 -1.043736112
C -13.117432055 -16.320398502 -0.724850005
C -16.76576789 -10.962705719 -3.186211983
C -16.261541282 -26.914172382 1.708545009
C -17.787183637 -9.929006425 -4.898248609
C -16.664571377 -10.360413519 -7.956920472
C -19.184329537 -13.938892853 -7.535876221
C -23.137117673 -18.122272298 6.288381568
C -12.7585331 -14.367915061 -1.815399444
C -15.884651029 0.252944432 -4.94760946
C -15.589866212 -12.504681711 2.824395567
C -14.292326252 -15.851226747 -1.119880016
C -21.241105409 -16.620150981 5.339757044
C -18.046904716 -15.842836773 -7.318351586
C -15.289931577 -9.347647816 -4.490603743
C -23.811436682 -17.814315373 7.601839777
C -21.578892149 -19.734205414 10.704450555
C -8.721012615 -17.017997019 -2.767989551
C -16.54487998 -4.632849995 -5.701524261
C -15.133128194 -19.770551032 3.121510257
C -19.863414695 -16.393595397 5.618538009
C -17.278979359 -5.094046552 5.803666555
C -18.829554513 -15.945286306 -8.515282856
C -22.889345251 -11.986915362 -6.628446854
C -15.577560738 -19.827728286 5.243526262
C -15.128550804 -13.718262799 -2.385825302
C -12.922137198 -9.787218729 2.121910295
C -20.747104347 -17.900612317 7.069893527
C -16.944193514 -9.01836871 -12.305942496
C -16.350663705 -4.391978367 -3.448958789
C -12.742890305 -8.886234498 3.12425369
C -14.585298064 -11.86926194 1.867138265
C -20.661244311 -14.62480259 -9.39469501
C -19.610854369 -8.647687213 -12.372285597
C -17.752082361 -5.019042485 -5.090825595
C -20.315965286 -15.019184462 -10.833797806
C -16.484136657 -19.954426984 3.201423964
C -14.397798834 -10.165603276 3.715390468
C -18.396358906 -17.288584591 7.572523955
C -13.882921023 -8.402920346 5.368412322
C -18.968202638 -22.064124637 -5.78334133
C -19.454694732 -22.941133873 -6.637355288
C -16.415456917 -16.500950887 3.634750126
C -20.51416075 -8.15068194 -11.549003745
C -20.797349362 -7.85926376 -3.703467836
C -16.22721773 -12.345024569 6.535080942
C -19.992166921 -21.831861655 0.909992674
C -18.596763121 -8.09929475 -10.452782916
C -19.551660488 -19.562142748 -5.248679135
C -18.068001558 -20.120680299 5.334141007
C -18.585858938 -5.135072968 -2.600646622
C -20.821340751 -21.179695808 -6.663798506
C -21.300803594 -22.915513351 -8.277495931
C -19.241754009 -19.517830013 4.429421187
C -18.116198733 -5.932351267 -1.419544802
C -19.814156639 -18.449937051 -6.320828193
C -2.128310415 -25.790190029 8.181500314
C 2.111375786 -21.119026075 -2.069003789
C -6.260144655 -22.764034204 -5.086732159
C -2.719249502 -25.465638881 9.556826327
C -5.147218915 -23.489516472 -4.276449127
C -20.43188865 -7.460509079 -9.040710411
C -20.719950317 -8.675040335 -8.118463117
H -7.694234019 -10.241013278 -12.156990749
H -11.58939272 -21.041989622 -4.67857766
H -6.422719775 -10.305250876 -13.407583776
H -15.680187266 -13.181957136 -11.458511282
H -11.172546565 -11.979828403 -9.68163067
H -12.951373441 -21.952671863 -4.090702523
H -10.873940175 -9.385551322 -9.520702418
H -9.360967508 -23.42479882 -0.516280348
H -7.683926472 -3.583016802 12.82453301
H -8.000812145 -11.100511035 -13.640506435
H -12.179071339 -17.426363804 -6.686638909
H -13.397776441 -15.22412459 -10.373419488
H -2.025596114 -25.800350924 0.817631353
H -8.815450999 -9.498420051 1.45531748
H -0.703462658 -24.847003506 0.212683871
H -8.718618076 -21.624700582 8.461618653

H -10.038611339 -11.127940669 -0.343873698
H -12.035935937 -7.248645946 -3.024645072
H -10.368095663 -18.693312612 5.721675978
H -13.664115502 -11.290528692 -11.642145286
H -7.146204549 -22.629017242 4.878515607
H -9.276868118 -11.87647104 -5.365185422
H -5.93585655 -24.578992167 3.529202394
H -6.291456313 -23.29335239 -0.053191224
H -13.664118118 -17.231899023 -7.539355216
H -0.925933458 -26.537979508 -0.364787491
H -9.8260512 -24.997482582 0.558060094
H -4.080452678 -23.145978231 1.374436055
H -0.438907188 -25.225096247 4.114224031
H -8.842903954 -12.204828422 -0.987953337
H -12.15352291 -17.361389849 -8.485507851
H -7.381742382 -25.724304614 3.883176376
H -9.302911543 -1.549451463 12.436980919
H -9.557924361 -7.639471682 -6.361467817
H -10.655990343 -1.929825857 11.38342761
H -10.475728245 -20.005404851 8.307886341
H -10.187109865 -15.088110194 -7.053873892
H -11.036298427 -23.752088655 0.251056698
H -6.2193022 -23.674116743 1.570724106
H -4.89516402 -9.793663682 -10.944416163
H -8.597300508 -20.5382897 6.97680374
H -10.730022399 -14.239873825 -4.094995601
H -5.690951805 -12.520366745 -12.417047599
H -9.140255747 -24.908838591 2.690671844
H -4.117965965 -25.995073487 -1.114132422
H -10.633653909 -12.713866677 -5.894840906
H -9.169841817 -0.994176445 10.810658996
H -2.490873836 -12.412142907 -2.008415954
H -7.398937711 -12.90990725 -11.774557564
H -1.779471009 -13.451440491 -0.690543313
H -9.376732752 -19.350515054 9.567154732
H -7.754203467 -16.254413625 -8.047723137
H -4.029079853 -24.966013449 1.320528057
H -6.207794881 -25.024129229 5.167031482
H -8.578799495 -10.249335577 -2.547276622
H -7.497450091 -20.324055688 8.326230199
H -1.417305485 -20.026255118 1.863188226
H -4.932887449 -14.124200551 -6.572232232
H -1.514031464 -27.147700082 5.998670216
H -2.705953677 -18.825235578 2.432614928
H -3.570447995 -13.564396436 -1.289145782
H -6.259123519 -25.060939949 0.44988346
H -10.023085228 -12.7275741 -3.164715941
H -4.418636147 -8.552365254 -8.838692501
H -8.876385471 -9.205402635 -1.14193992
H -5.785128915 -8.794251812 -7.788680009
H 0.733494862 -26.91452308 1.440255422
H 0.30015405 -25.325554395 2.079985744
H -3.877433351 -18.253816047 -1.635516754
H -11.17497162 -4.7588904 -3.855784922
H -7.417524339 -9.921753735 -1.335525788
H -9.010393227 -14.077286911 -3.762723256
H -2.922214871 -20.073458168 -0.394088137
H -7.029298949 -13.266579946 -9.447430299
H -4.613023415 -9.637824079 -3.782827007
H -6.977964121 -12.797033698 -5.55296802
H -1.572644246 -18.420018055 0.979846457
H 0.50821115 -15.990241773 -0.320781001
H -2.281636347 -25.502164954 -3.034557915
H -5.295529586 -15.828077688 -6.849383284
H -5.364212374 -14.778515198 -8.332978559
H 0.842998246 -18.207818194 1.850496401
H -0.189453291 -14.976216831 -2.028475134
H -4.365573973 -6.56793389 -4.01374028
H -10.128378458 -4.694610668 -7.178889845
H 1.868010761 -16.75324986 1.554030104
H 1.747602264 -12.477614117 9.105538117
H -0.394479865 -21.933515672 5.261753724
H -8.56288548 -5.584356009 -6.637248146
H -1.462470951 -22.164328839 3.863921625
H -6.57577819 -11.628566363 -7.440145385
H -3.5494176 -8.697006986 -2.654288767
H -4.4028139 -9.624294569 -7.369645228
H -4.70961277 -8.39887157 -0.494120603
H -2.03468865 -14.153275971 6.843097228
H -5.889966931 -18.182375201 -7.987207314
H -4.170601558 -18.565196237 -8.452215141
H -2.740223252 -29.286042946 5.807169611
H -4.818373119 -18.011310615 -6.595385088
H -3.656623711 -20.809160267 1.146054258
H -2.815020199 -7.375930311 -4.330090737
H -2.173601484 -22.324494504 -0.215279431
H 1.812072671 -10.78318857 9.650473204
H -9.345955417 -4.242774475 -5.565805488
H -3.640941337 -13.724495538 6.090683957
H -0.880525687 -17.041695825 0.014352355
H -1.481322974 -28.743025066 1.995897058
H -4.153584561 -14.619467569 -3.396994435
H -3.622664666 -11.785894753 -0.118589367
H -6.000284323 -16.491288545 -1.31704359
H -4.049781466 -12.832659722 1.311823496
H 0.408810429 -12.145039409 7.034524295
H -1.991273774 -21.064888128 5.225511596
H 0.080072785 -14.393086648 3.052935614
H -4.014963922 -23.106867979 4.728254197
H -2.105501218 -13.366967554 2.760361174
H 1.47149206 -14.46135767 1.896906362
H -0.333814736 -15.957270551 1.210063102
H -3.391434929 -13.361665987 7.691510412
H 1.137615962 -11.891709402 10.756762451
H -1.985802837 -30.421036411 4.578482288
H -4.176378502 -7.624736877 -5.34767496
H -3.477919761 -29.447393627 4.239747411
H 4.081279497 -17.790651732 0.731388081
H 1.771840284 -14.37116312 3.569219548
H -1.062290263 -21.842188572 -2.806588957
H -0.356761116 -16.581876021 -4.326623106
H -4.234796648 -11.007853627 1.347820642
H -3.221565939 -11.245565595 6.283962348
H -7.051281512 -7.175092165 0.233835823
H -0.680414522 -23.517780214 -3.466395061
H -5.475011316 -19.289820018 2.017679146
H -4.783267045 -21.106009404 -9.198168765
H -2.982001392 -17.498341896 -5.350548663
H 0.392054876 -19.12821248 -0.9740583
H -0.200534798 -23.028416736 -1.72197872
H -1.464826568 -10.92668174 10.758104246
H -6.876193978 -7.13173455 -4.007999217
H -4.032103321 -16.149257349 -5.628495576
H -5.937849075 -20.01607579 -5.189312818
H -8.40917536 -17.877183984 0.692486746
H -3.227676759 -10.798106705 8.56709577
H -1.831505183 -11.865699677 5.460369377
H -0.523613204 -23.728562668 6.931536779
H -4.623194523 -25.153536203 6.676095553
H 0.609185996 -12.269983094 3.829601136
H 1.729198402 -11.980740091 2.602155334
H -7.784217344 -17.284816604 2.255137791
H -1.799114755 -9.980600779 0.109084695
H -7.751334814 -16.277111121 0.742229308
H 4.552382427 -20.134140982 -0.950895047
H -2.788714111 -16.751269435 -7.710439913
H 0.809949549 -10.76235519 0.809460401
H -9.319629904 -6.213516566 -1.224836508
H -1.342474683 -16.020334922 -6.924092739
H -8.945007607 -5.870195011 -2.969346517
H -5.346770154 -23.315883682 -7.823383932
H -2.678543128 -14.922894555 -7.166681869
H -8.246537256 -4.91540611 -1.721268797
H -8.31187441 -23.934216466 5.323921897
H -17.426933266 -22.00067185 -8.819674252
H -14.927238288 -19.167789517 -8.56664657
H -16.966190094 -20.938002285 -7.532684365
H -11.103461227 -15.093666537 -12.843642519
H -13.426669834 -26.074880199 -10.303440955
H -12.006571627 -24.88288538 4.199858739
H -9.702950249 -3.491249429 9.131944677
H -12.027455156 -16.215943353 -10.704495886
H -10.206483549 -9.736267533 -4.167959522
H -13.641778116 -16.990365785 -10.841913363
H -11.485152213 -21.181177452 -2.055146877
H -12.141556548 -9.475723104 -4.108151474
H -12.560104922 -23.520079507 -11.106563077
H -7.086389248 -13.321721922 0.73585679
H -15.146875237 -26.302328432 -10.863691751
H -12.738226723 -9.413593088 -9.416914657
H -9.104459941 -21.3649252 0.759115511
H -8.05221859 -9.705335163 4.411061491
H -11.338414122 -20.255697651 -6.608730572
H -7.77753189 -21.128825833 3.287700804
H -7.936181035 -17.219313929 9.166975267
H -9.506081902 -16.410127876 5.110749249
H -13.203666523 -20.252490475 -4.508929331
H -13.283093658 -22.187147994 4.961140735
H -11.955865919 -9.922075092 -10.935426857
H -11.810095024 -10.825345511 -6.174457415
H -13.22603645 -18.062733299 -3.46230856
H -6.930895983 -8.824384003 3.151908227
H -10.288789654 -10.142745485 -6.563041154
H -13.238539101 -27.527224192 -12.047089354
H -11.751345066 -11.354746076 -8.163737928
H -12.777304217 -26.122494809 1.703631956
H -16.480537335 -20.404484998 -9.260961472
H -15.335553451 -14.814329209 -8.040521024
H -13.119490957 -20.451301351 -1.876081659
H -6.963097821 -5.804691928 11.461170445
H -10.8489646 -13.726995442 -15.02820842
H -7.876003249 -6.770773182 9.341133939
H -14.921107692 -22.43872011 5.104222924
H -15.248681687 -22.704289386 -7.612001616
H -13.044522102 -12.652230652 -15.836213179
H -14.17003268 -21.169791901 -11.819954729

H -12.772190656 -22.543808844 -7.161625972
H -6.316878026 -12.431903599 3.249854389
H -6.286441197 -10.216310859 4.167157892
H -11.591692933 -9.146911408 -6.777894103
H -20.030958647 -12.143055523 -4.962535533
H -14.829723026 -22.510370273 -9.316161634
H -12.655438636 -25.834974133 -12.446164696
H -10.135275959 -14.646215469 -1.481727445
H -13.341313451 -13.508046412 -7.981986193
H -14.11768353 -26.386627165 -13.35693148
H -17.347003062 -14.816077131 -4.917547741
H -16.603066843 -13.698900116 -8.506939323
H -13.954197165 -23.577997643 6.08924069
H -8.502428908 -5.657140468 8.024568998
H -10.318863097 -16.316657005 -0.641723939
H -16.154886865 -15.293381711 -9.571396864
H -11.73870857 -12.865772585 -17.059445594
H -15.304242757 -15.943368185 -12.990380977
H -10.669325551 -6.986861376 8.360763966
H -6.9861503 -15.354770258 5.953699503
H -16.404965114 -14.171815665 -6.203406681
H -7.844755322 -14.345633665 7.087800797
H -12.44507016 -19.936923527 4.272994348
H -20.922757196 -10.806411088 -4.428731468
H -14.506562867 -2.531853352 -5.108617083
H -6.548810664 -15.484999759 7.725953708
H -15.861629471 -7.235483473 -5.319301849
H -10.915922548 -15.014421091 -16.234967049
H -16.890665928 -20.96135676 -11.39972361
H -18.813314984 -11.858972958 -2.463944676
H -10.830919817 -13.602462093 0.629329992
H -16.02610032 -24.79168559 0.807149919
H -16.714403046 -24.40951399 -11.588065918
H -11.359205081 -16.132810478 -3.639434766
H -12.99115334 -18.30291716 4.523360556
H -13.285534549 -13.83652287 -17.162645546
H -13.045292951 -19.530973436 5.935622944
H -17.52830125 -13.084602527 -5.299383877
H -15.87722649 -23.515941989 3.186511175
H -13.908778133 -4.241743621 -5.625456986
H -9.704259346 -7.640176947 9.783852876
H -20.068917038 -10.825268334 -5.961934535
H -9.40372124 -19.563218512 -2.06254667
H -14.348310474 -7.59531109 -4.308913703
H -23.610955994 -17.574077311 5.407755109
H -13.825027524 -3.618928987 -3.907211498
H -12.804323712 -17.046286117 0.042354328
H -17.043414359 -27.267041061 0.945055105
H -14.396494583 -15.528401606 -15.481309802
H -10.715529199 -6.171778989 9.882570514
H -16.368815869 -11.754343888 3.136646241
H -16.739424214 -21.555559587 -13.259549497
H -21.898875283 -16.227496397 4.55479015
H -14.726018412 -25.926264949 0.43833642
H -15.839498578 -7.47136198 -3.530575078
H -11.284639261 -15.179865234 1.241394569
H -19.56685411 -12.855004363 -7.347518301
H -15.894220404 -11.154288657 -2.534727072
H -17.618696165 -22.469095229 -12.040470725
H -23.158983178 -19.188841074 6.083592837
H -15.041945007 -13.01912415 3.628858376
H -9.646505335 -14.920035352 1.08836724
H -15.970396014 -13.373564232 2.3088461
H -24.018332906 -16.822795672 7.869587454
H -17.938398537 -9.285624698 -5.811828001
H -16.215785672 -4.628891219 -6.73654327
H -12.190397246 -13.56431889 -2.367050183
H -14.43237644 -19.749602274 2.246088818
H -17.239178559 -16.517414148 -6.86861104
H -16.832946932 -26.521307314 2.57524343
H -15.572893956 -27.693457045 1.930556295
H -16.783399645 -10.099411758 -12.252289586
H -23.273453783 -18.230829014 8.477412069
H -15.320752229 -16.155493853 -1.028559289
H -15.736654435 -13.235217162 -1.697491828
H -12.415153235 -10.005503285 1.233881078
H -19.128329533 -15.841131826 5.117741033
H -14.555832388 -9.712612525 -3.739867325
H -8.778061847 -15.953963588 -2.764783502
H -21.438674626 -15.294333998 -9.079018536
H -19.759958919 -9.051903383 -13.347062649
H -14.933194507 -9.650591841 -5.538137222
H -8.154887243 -17.393476458 -3.6816801
H -8.174606696 -17.361811071 -1.833852917
H -17.024613721 -8.77246597 -13.369396036
H -15.650406179 -19.681125766 6.3375502
H -11.952080579 -8.150721276 3.158540716
H -21.146669436 -14.900785091 -11.505952239
H -14.788741263 -7.781334953 5.533641891
H -24.818132462 -18.310442059 7.432181706
H -15.93075049 -4.218055294 -2.496675469
H -16.044065345 -8.497239841 -11.808788196
H -14.465162651 -13.03301765 -2.971876196
H -18.040521016 -22.069567198 -5.322201714
H -19.067196371 -23.815034764 -7.192606178
H -17.519774004 -17.629698541 6.99422156
H -15.074375136 -11.481917714 0.95167461
H -18.911377441 -16.637328297 -9.29611812
H -20.84590563 -18.571223855 7.95419432
H -15.786951116 -14.336957254 -3.041645643
H -18.093212531 -16.212755256 8.016735659
H -13.777708472 -12.515218714 1.424697829
H -20.999494341 -13.631430976 -9.375892427
H -15.088156658 -10.729363021 4.330659545
H -18.684918541 -5.576638607 -5.396770992
H -19.59179955 -14.260169126 -11.118799741
H -12.95182622 -7.844388019 5.424459532
H -19.842652037 -15.975839994 -10.817520021
H -17.21259065 -20.244658959 2.441445021
H -18.527243419 -19.465799699 -5.039355057
H -13.972106695 -9.101607372 6.199457115
H -21.550152646 -8.097909468 -11.82880526
H -18.732950947 -17.976115984 8.406886466
H -21.715299339 -22.112972644 -8.905417163
H -18.077241887 -21.206048461 5.60260036
H -18.797652684 -18.621028941 4.068553845
H -17.897757215 -7.940055072 -9.642768544
H -18.114951694 -7.058930123 -1.836453219
H -18.854511838 -4.148628348 -2.323390445
H -20.78424803 -23.772697677 -8.777627713
H -21.60860091 -9.355510949 -8.365010339
H -20.14240444 -19.389702179 -4.355331298
H -19.319043886 -17.523112417 -5.927362918
H -18.033680675 -19.591020466 6.315054433
H -20.156427996 -19.488509565 4.996300969
H -19.497900994 -5.53744366 -3.023570098
H -19.487172615 -18.593339018 -7.338912231
H -18.994694548 -5.799765416 -0.590809137
H 2.896248093 -21.276715609 -2.774076561
H -6.855284834 -22.081817109 -4.431867843
H 1.107070311 -20.956219387 -2.534287067
H -1.013055719 -25.855844992 8.256468369
H -2.290750936 -26.765038882 7.715839014
H -3.016064478 -26.314532196 10.130239038
H -1.866520471 -25.108708184 10.142533212
H 2.079135413 -21.891516964 -1.335808705
H -4.635177531 -22.974640438 -3.543416546
H -6.882606179 -23.516951524 -5.556560907
H -4.374384741 -23.905841219 -5.032760188
H -3.614918594 -24.974961167 9.540270123
H -5.631426318 -24.421376093 -3.868550656
H -21.733331667 -20.659893412 -6.855547183
H -19.625024864 -6.85657839 -8.573773492
H -22.169629235 -23.452929251 -7.871337176
H -19.363479507 -20.133675715 3.550658878
H -17.042440333 -5.742715707 -1.016491096
H -20.79481866 -8.318182245 -7.063837113
H -20.830257041 -18.191119203 -6.469316218
H -21.280708638 -6.693610512 -9.273989778
H -19.761787586 -9.020011401 -8.289857038
N -8.79832727 -3.053325246 11.020839661
N -8.260256404 -11.398004455 0.686969231
N -9.261441197 -18.31209749 7.686727636
N -8.466244908 -23.135949385 3.391805581
N -9.535791727 -23.21222379 1.476744288
N -10.131793122 -13.000703221 -12.70317313
N -10.971542344 -7.9366701 -4.826486534
N -11.280777139 -21.255037655 -13.749304465
N -10.314303288 -17.092812582 2.808249974
N -12.358204411 -4.222606523 -8.424834041
N -7.032562803 -14.629205766 -1.576729062
N -6.293424872 -11.535411215 -10.669349653
N -8.372856808 -8.810417818 -8.185385104
N -8.873688788 -13.656415665 -6.343163287
N -10.21179535 -5.947933554 -5.428796297
N -3.387615815 -8.481628791 7.927746029
N -0.806321197 -26.875792857 2.787150405
N -5.481384721 -10.477608725 -8.933272293
N -3.674836059 -24.012623366 -0.514501494
N -7.015532259 -14.520859087 -6.986700692
N -4.584890861 -13.448433054 3.979707982
N -1.741789692 -28.313145268 4.120701013
N -2.161075043 -14.420667465 -2.574403034
N -9.185692859 -18.276500899 -8.635366326
N -4.326862101 -18.893569612 0.337552719
N -2.099013571 -23.577035637 -1.946718529
N -3.450293058 -8.62155486 1.059574526
N -5.602206119 -8.143815115 -2.482537593
N -4.709174957 -21.588351902 2.981915331
N -6.464999426 -26.246432994 -5.810976493
N 0.771173056 -3.574431876 -11.305709276
N -0.109795036 -11.589521243 9.057319323
N 2.016793714 -18.297846889 0.207583418
N -2.101191035 -23.125653534 5.6742238
N -2.480375977 -15.6804956 -4.389585311
N -6.17676326 -17.618913816 0.724042582

N -1.646121035 -11.541935317 7.524432297
N -5.100798756 -20.049099086 -7.23520415
N -2.207630867 -11.720989062 1.377595414
N -1.603782638 -18.670370472 -2.53329466
N -0.193804657 -12.262525229 1.959702372
N -7.329466721 -6.913734743 -1.895039982
N 0.399827745 -10.361896839 5.417788436
N -2.488913802 -24.756265985 7.060671671
N -5.439214671 -16.25702651 9.457673586
N 2.395044335 -19.940708733 -1.219374839
N -5.61461399 -21.941812544 -6.15849803
N -13.058047942 -19.252204619 -7.586674105
N -13.218275942 -12.072799383 -9.610180025
N -7.558815155 -10.729798969 2.652975035
N -13.507876684 -24.542646985 -1.907117107
N -14.075435519 -21.050183387 -8.129291363
N -13.096895956 -15.694305144 -12.459439535
N -8.397290888 -4.918012835 9.941806731
N -14.76903021 -13.645159423 -9.559760794
N -8.344183414 -16.409013784 7.111366661
N -8.405183496 -2.464953311 -2.590833533
N -9.398194424 -6.81893225 2.876519093
N -13.072050333 -11.86087513 -4.68495846
N -14.546569283 -24.48737733 -11.331678208
N -13.932899409 -23.99634657 3.969992919
N -12.432337908 -15.086904014 -14.455911912
N -11.529555517 -19.261564581 -2.729325242
N -18.41599365 -13.884125854 -1.113809075
N -14.58309 -25.03786 2.19810
N -15.59286 -22.64282 -11.95694
N -12.23354 -11.64376 -0.70913
N -15.23033 -18.94488 -12.21735
N -18.75380 -10.71606 -4.30373
N -16.22866 -0.86729 -4.74622
N -12.16955 -15.30773 -1.08487
N -10.02020 -17.65367 -2.86743
N -18.27054 -14.61392 -6.70501
N -16.65952 -11.54608 -8.03202
N -16.62683 -10.12297 -4.27960
N -14.61671 -19.57933 4.37238
N -15.66498 -4.27906 -4.68955
N -14.08824 -14.61700 -1.76543
N -21.74226 -17.58410 6.24951
N -16.74445 -6.04696 6.22342
N -19.53241 -14.76719 -8.52099
N -22.64186 -20.07427 11.15883
N -19.67273 -17.15809 6.75120
N -13.92453 -10.68439 2.60064
N -17.20430 -16.70613 4.48458
N -13.70935 -9.06691 4.07541
N -21.86658 -11.90746 -7.29167
N -18.33764 -8.66260 -11.68336
N -20.57104 -7.47206 -4.83605
N -16.72489 -19.96759 4.60639
N -17.56587 -4.94503 -3.72264
N -15.51414 -11.43017 6.29439
N -19.89759 -20.93505 -5.73044
N -20.55336 -22.40167 -7.18180
N -19.90760 -7.84137 -10.35699
N -20.81622 -21.35307 0.18619
S -10.68198 -23.97774 -13.48888
S -13.29535 -6.58472 -7.18565
S -11.87241 -19.03934 1.34710
S -7.17435 -0.35540 -1.01966
S -5.88454 -12.18770 -2.58310
S -7.38103 -9.67970 -5.62321
S -9.53897 -14.11302 -10.22357
S -3.22944 -15.59650 2.77521
S -4.42323 -25.84949 -7.67788
S -5.74568 -6.95129 7.61350
S -6.17909 -20.62077 5.24010
S -5.01968 -7.02159 2.83840
S -8.29827 -19.33519 -6.05400
S -3.69158 -14.06046 10.04324
S 2.33915 -4.84237 -9.28106
S 2.19325 -9.04260 3.62298
S -2.98023 -20.55662 -4.16712
S -14.63206 -22.59191 -0.02656
S -17.81523 -16.18635 -2.76493
S -12.95157 -14.58380 -5.47476
S -10.56913 -7.95403 0.51918
S -20.11822 -19.28359 10.12694
S -16.46212 -17.64407 -9.98774
S -24.03874 -12.19546 -5.49129
S -16.53982 -8.72774 -8.00581
S -13.45894 -9.15381 -1.22151
S -15.69049 1.87770 -5.18420
S -18.06043 -3.71414 5.13949
S -18.94251 -22.44694 2.10762
S -17.44341 -13.44624 6.78220
S -20.95775 -8.24708 -2.05594
S -15.32189 -16.15082 2.41426
S -7.76773 -18.80590 -4.61664
O -7.18193 -17.47859 -5.58389
O -6.75793 -18.85680 -3.30009

Geometries and energies of SO_2 molecule, $[SCN]^-$ anion and anion-SO_2 adducts ($[NCS{\cdot}SO_2]^-$ and $[SCN{\cdot}SO_2]^-$):

SO_2

$E_{\rm tot}$(RI-BLYP-D3(BJ)/def2-TZVPP) = −548.7119120

S 0.000000 -0.000000 0.174472

O 0.000000 -1.259582 0.917671

O 0.000000 1.259582 0.917671

$E_{\rm tot}$(B3LYP-D3(BJ)/def2-TZVPP) = −548.6029282

$E_{\rm tot}$(CCSD(T)/CBS//B3LYP-D3(BJ)/def2-TZVPP) = −548.0815700

S 0.000000 0.000000 0.182191

O 0.000000 -1.238742 0.913811

O 0.000000 1.238742 0.913811

[SCN]$^-$

E_{tot}(RI-BLYP-D3(BJ)/def2-TZVPP) = −491.1471375

S 1.840458 -0.000000 0.000000

C 0.173073 0.000000 -0.000000

N -1.013531 -0.000000 0.000000

E_{tot}(B3LYP-D3(BJ)/def2-TZVPP) = −491.0678994

E_{tot}(CCSD(T)/CBS//B3LYP-D3(BJ)/def2-TZVPP) = −490.5758235

S -0.000000 -0.000000 -3.262674

C 0.000000 0.000000 -1.602481

N -0.000000 -0.000000 -0.429976

[NCS·SO$_2$]$^-$

E_{tot}(RI-BLYP-D3(BJ)/def2-TZVPP) = −1039.8969089

N 0.414423 1.746896 -3.032803

C -0.614095 1.716268 -2.454019

S -2.076509 1.668760 -1.649534

S -1.593892 -0.560155 -0.077801

O -0.765420 -0.046317 1.039443

O -2.990207 -0.915555 0.285280

E_{tot}(B3LYP-D3(BJ)/def2-TZVPP) = −1039.7061239

E_{tot}(CCSD(T)/CBS//B3LYP-D3(BJ)/def2-TZVPP) = −1038.6835077

N 0.396362 1.713325 -2.982639

C -0.624209 1.691535 -2.418153

S -2.087389 1.652197 -1.625207

S -1.590080 -0.540016 -0.108497

O -0.761433 -0.026195 0.974980

O -2.958952 -0.880948 0.270082

$[SCN{\cdot}SO_2]^-$

$E_{\rm tot}$(RI-BLYP-D3(BJ)/def2-TZVPP) = −1039.8935382

S 0.163613 1.730856 -3.603920

C -0.830348 1.628987 -2.308184

N -1.563223 1.573975 -1.365387

S -1.549605 -0.366801 0.009077

O -1.252951 0.232876 1.328263

O -2.938230 -0.843441 -0.170952

$E_{\rm tot}$(B3LYP-D3(BJ)/def2-TZVPP) = −1039.7032387

$E_{\rm tot}$(CCSD(T)/CBS//B3LYP-D3(BJ)/def2-TZVPP) = −1038.6824159

S 0.149014 1.775235 -3.585662

C -0.831758 1.607526 -2.296092

N -1.548745 1.499084 -1.363894

S -1.554637 -0.368157 0.007927

O -1.254914 0.252154 1.288456

O -2.929704 -0.809389 -0.161838

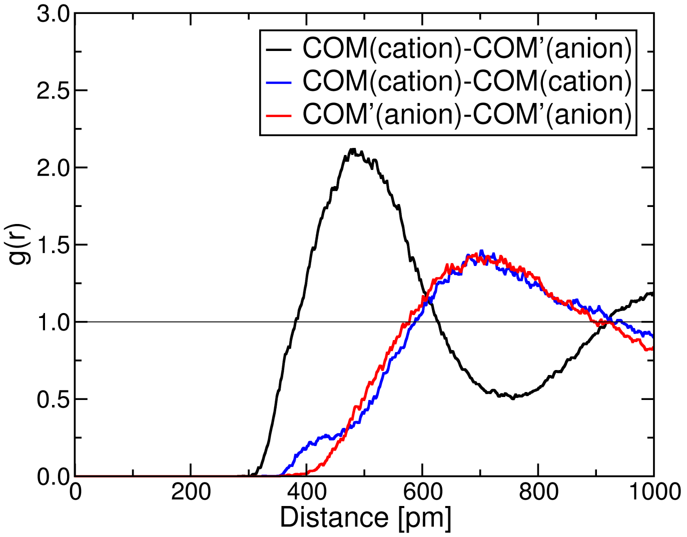

Figure S1: Radial distribution function between the center of masses of cation and anion (black), cation and cation (blue), anion and anion (red).

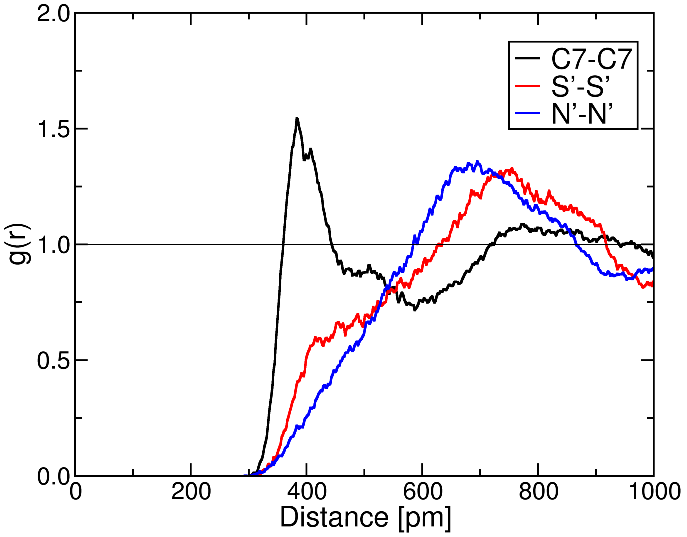

Figure S2: Radial distribution function between the selected atoms of cation and anion (see Figure 2 in the article for atom labels).

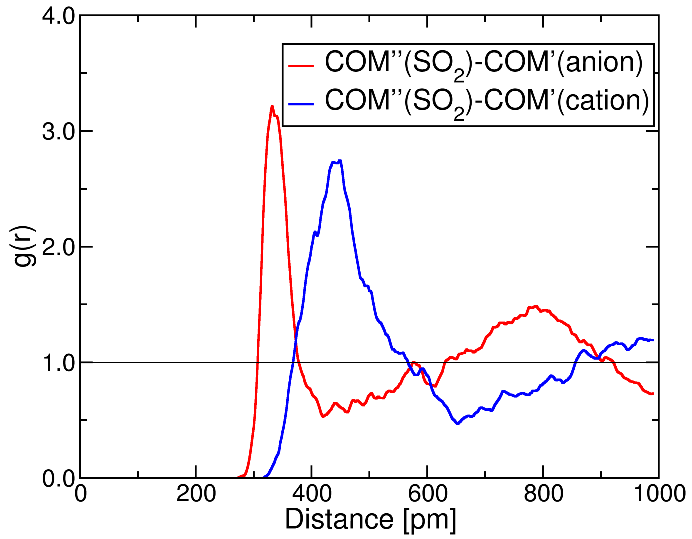

Figure S3: Radial distribution function between the center of masses of SO_2 and anion (red), SO_2 and cation (blue). The value of integrals in the first minimum are 1 for blue curve and 5 for red curve.

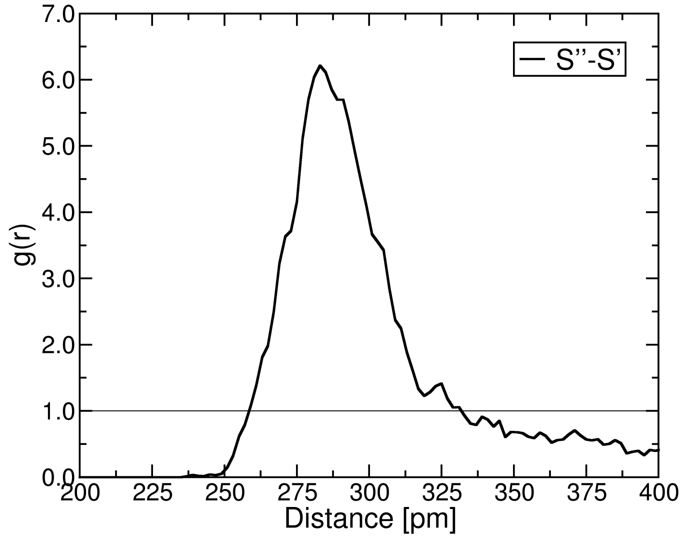

Figure S4: Radial distribution function between the sulfur atoms of SO_2 and anion.

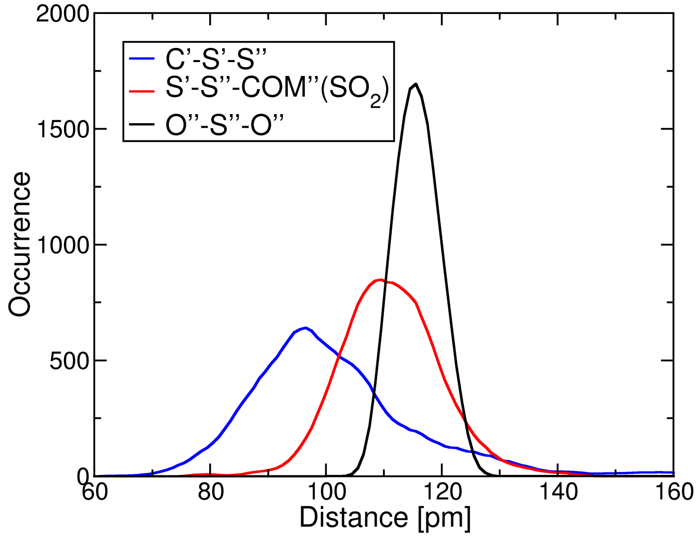

Figure S5: Angular distribution function between center of masses and atoms of SO_2 and the closest anion.

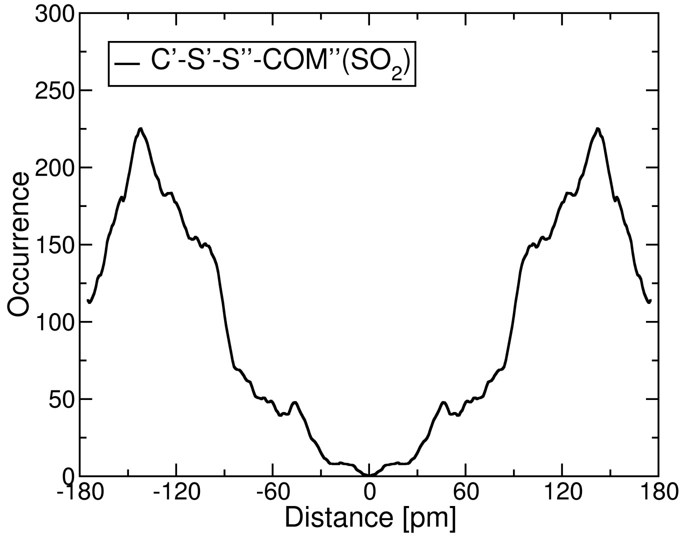

Figure S6: Dihedral distribution function for dihedral angle between anion and SO_2.
